# Valerianate of Zinc

**Published:** 1847-11

**Authors:** 


*Valerianate of Zinc.*—Dr. Neligan gives the following account of the mode of preparing, and medical properties of this new remedy.

*Preparation.*—"Take of the bruised root of valerian, two pounds; water, eight pounds; sulphuric acid, three ounces one drachm:

macerate for two days, and distil until the liquid no longer reddens bibulous paper. Let the distilled liquor be then exposed to the air for a month, at the end of which time, put it into a matrass, with half an ounce of recently precipitated, perfectly pure, hydrated oxide of zinc, and digest for from eight to ten hours on a sand-bath, heated to 176 degrees F., stirring occasionally. Filter the warm liquor, evaporate it to three-fourths of its volume, pour into porcelain capsules, and expose to the heat of a stove until crystals are formed, which are to be dried with filtering paper.

*Therapeutical Effects.*—Valerianate of zinc is a tonic antispasmodic of much power, and as such, is peculiarly adapted for the treatment of neuralgic affections, which are so generally dependent on loss of tone in the system. It has been found especially useful in the treatment of facial neuralgia and of vertigo; but I have seen it prove equally beneficial in most of the Protean forms of hysterical neuralgia. In short, I look on it as one of the most valuable modern additions to the materia medica; and I fully agree with the observations of Devay, that the chemical combination proves much more beneficial than the oil of valerian and oxyde of zinc prescribed together.

"*Dose and Mode of Administration.*—The dose of it is from three-fourths of a grain to one grain twice or three times a day; it may be prescribed in the form of a pill made with a little mucilage or conserve of red roses, or in solution in orange-flower water, or in distilled water flavored with syrup of orange-flowers. The compounder must bear in mind, that the crystals of valerianate of zinc do not dissolve readily in cold water, floating on the surface, in consequence of their lightness; they should, therefore, be first incorporated with a few drops of water in a mortar.

"*Incompatibles.*—All acids; the soluble carbonates; most metallic salts; and astringent vegetable infusions or decoctions."—*London Med. Gazette, May* 1847, *from Heller's Archiv.*, 1846.

